# Hypertension and Associated Factors in Rural and Urban Areas Mali: Data from the STEP 2013 Survey

**DOI:** 10.1155/2018/6959165

**Published:** 2018-01-21

**Authors:** Hamidou Oumar Bâ, Youssouf Camara, Ichaka Menta, Ibrahima Sangaré, Noumou Sidibé, I. B. Diall, Souleymane Coulibaly, Maiga Asmaou Kéita, Georges Rosario Christian Millogo

**Affiliations:** ^1^CHU Gabriel Touré Bamako, Bamako, Mali; ^2^CHU Kati, Kati, Mali; ^3^CHU Point G, Bamako, Mali; ^4^CHU Mère-Enfant, Bamako, Mali; ^5^CHU YO, Ouagadougou, Burkina Faso

## Abstract

**Background:**

Our study aims to estimate hypertension (HTN) prevalence and its predictors in rural and urban area.

**Methods:**

We conducted a cross-sectional population-based study involving subjects aged 15 to 65 years. Collected data (sociodemographic, blood pressure, weight, height, and blood glucose) were analyzed using SPSS version 20. A logistic regression was conducted to look for factors associated with HTN.

**Results:**

Mean was 47 years. High blood pressure (HBP) prevalence was 21.1 and 24.7%, respectively, in rural and urban setting. In rural area age group significantly predicted hypertension with age of 60 years having more-than-4-times risk of hypertension, whereas, in urban area age group, sex and body mass index were predictors with OR: HTN raising from 2.06 [1.24–3.43] for 30–44 years old to 7.25 [4.00–13.13] for 60 years and more using <30 years as reference. Female sex was protective with OR of 0.45 [0.29–0.71] and using normal weight as reference OR for overweight was 1.54 [1.04–2.27] and 2.67 [1.64–4.36] for obesity.

**Conclusion:**

Hypertension prevalence is high and associated factors were age group in rural area and age group, female sex, and body mass index in urban area.

## 1. Introduction

Hypertension (HTN) as leading risk factor for cardiovascular diseases has been largely described by many authors in the world [1–4]. As in other parts of the world HTN is a public health problem of growing concern in Low- and Middle-Income Countries (LMIC) [5, 6] and particularly in Africa [7, 8]. Newer African data on prevalence from community-based studies are high and above 25% [9, 10] and up to 53% [11]. Many factors have been described as associated with HTN [2, 12, 13].

Published data and mostly those from population-based studies are either old or rare in Mali. Prevalence data based on a 2002 conducted survey found 26% in the urban setting Bamako [14]. Recent data are therefore needed as well in urban as in rural areas. Our study aims to estimate hypertension prevalence and its risk factors in some rural and urban areas based on 2013 step survey data.

## 2. Materials and Methods

We conducted a cross-sectional population-based study, whose data stemmed from the 2013 STEPS-Survey in urban and rural areas. This approach has been described by the World Health Organization (WHO) [15].

### 2.1. Sampling and Data Collection

The study sample is based on the last STEPS-Survey which was conducted in 2013 with 2102 subjects aged from 15 to 65 years of whom 1543 (73.4%) in urban and 559 (26.6%) in rural areas.

It was conducted in administrative units known as communes and within each commune in towns/villages.

A two-stage cluster sampling method was used to select subjects from urban and rural areas. First clusters were obtained among communes in the three involved regions and second clusters from quartiers within these communes. Households were then randomly selected and all eligible adults in the household were interviewed and underwent physical exam and measurements.

The sample size was calculated using the formula

(1) $$\begin{array}{c} n=\frac{Z^{2}pq}{i^{2}}\times d \end{array}$$

with *n* = sample size,* Z* = 1,96, *p* = 40% = 0.40, *q* = 1 − *p* = 0.6, *i* = 3% = 0.03, *d* = 2.

A total of 30 clusters as given in Table 1 were obtained.

### 2.2. Data Collection

The following data were recorded for each study participant:

- Sociodemographic
- 3 blood pressures and heart rate measures in 5 min interval, using their mean as systolic, diastolic blood pressure, respectively, SBP, DBP, and HR
- Fasting/postprandial glycemia
- Weight in kilogram (Kg) and height in centimeter (cm) allowing calculation of body mass index (BMI) in Kg/m^2^ as weight (Kg)/height (cm)^2^
- Waist circumference (WC) and hip circumference (HC) all in cm
- Waist-to-hip ratio (rWH) as WC in cm divided by HC in cm
- Mean blood pressure from 3 measures

For the filling of the survey formulary and performing measurements, medical teams were built and trained for the household visit (and second visit in case household members were not present). Each subject was interviewed and different measures were obtained according to guidelines of the WHO STEPS approach for chronic disease surveillance [15].

Interviews were performed by medical staff and focused on sociodemographic characteristics, lifestyle, cardiovascular risk factors, personal and family history of cardio-vascular diseases, or other chronic illnesses.

Height was measured without shoes to the nearest centimeter with subject stand on the footplate with back against stadiometer rule.

Weight was measured to the nearest 0.1 kg on an electronic scale with the subject wearing light clothing and no shoes.

Waist circumference (WC) and hip circumference (HC) were measured with a stretch‐resistant tape that is wrapped snugly around the subject, but not constricting.

WC was measured at the midpoint between the lower border of the rib cage and the iliac crest with the subject being light clothed.

HC was measured around the widest portion of the buttocks.

We used for blood pressure measurements a Frangly® aneroid sphygmomanometer with a medium and large cuff size and performed measures at rest, the subject being in sitting position on the right arm. The mean of the two blood pressure readings was used for each subject in this study. A third measure was performed in some cases if blood pressure values were borderline.

### 2.3. Definitions

Education level was graded as follows:

- Level 0: no school attending
- Level 1: school attending for 1–6
- Level 2: school attending for 7–9
- Level 3: school attending for 10–12
- Level 4: school attending for 12 and more years

Hypertension was defined as systolic blood pressure (SBP) ≥ 140 mmHg and/or diastolic blood pressure (DBP) ≥ 90 mmHg or self-reported use of antihypertensive drug irrespective of measured blood pressure [16].

Mean arterial blood pressure (mBP) was calculated with the following formula: DBP + ((SBP − DBP)/3).

General obesity was defined by body mass index (BMI) and further central obesity through the waist circumference (WC) [17].

BMI served to define weight disorders as follows:

- Underweight (UW): <18.5
- Normal weight (NW): 18.5–24.99
- Overweight (OW): ≥25.00–29.99
- Obesity (OB): ≥30.00

Based on waist circumference, OW was defined as WC ≥90 cm and ≥80 respectively for men and women and OB as waist ≥102 cm for men and ≥88 cm for women [18].

For waist-to-hip ratio (rWH) Men with a rWH 0.90–0.99 and women with a rWH 0.80–0.84 were classified as overweight, whilst men with a rWH ≥ 1.00 and women with a rWH ≥ 0.85 were classified as obese [18].

Diabetes was assessed with a glucometer (one-touch ultra Bayer®), fastened or postprandial with cutting values of 1.26 and 2 g, respectively, and the use of antidiabetic medicine.

### 2.4. Data Analysis

Statistical analysis was done using analytical software SPSS version 20. Results were expressed as either mean values (standard deviation) or proportions. *t*-test for continuous variables and chi-square or Fisher test analysis for categorical variables were used. Level of significance was set at 0.05.

After a prevalence estimation in rural and urban setting, hypertensive subjects were selected to compare them regarding sociodemographics and descriptives variables.

Finally a logistic regression was conducted to look for predictors of hypertension in rural and in urban setting using age group, marital status, sex, educational level, tobacco smoking, alcohol consumption, body mass index, waist circumference, and waist-to-hip ratio and diabetes as independent variables.

For logistic regressions the following references were used: <30 years, single, Female, unschooled, nonsmoker, no alcohol consumption, normal weight for BMI, WC, and rWH, no diabetes, and resting HR > 90/min

## 3. Results

From the 2102 study participants, 495 were hypertensive subjects of whom 118 (23.8%) resided in rural area.

Mean age of the sample was 47.78 ± 13.230 (47.00 ± 13.214 in rural and 48.03 ± 13.243 in urban area with*p* = 0.451). Height and Glycemia were slightly higher in urban areas with 167.60 versus 165.76 cm and 120.18 versus 116.23 mg/l with *p* value of 0.074 and 0.536. rWH was not significantly different with 0.91 in urban and 0.92 in rural area and *p* value of 0.634 (Table 2).

Weight, BMI, WC, and HC were higher in urban areas compared to those in rural areas, 75.47 versus 68.50 kg, 26.29 versus 24.83 kg/m^2^, 92.79 versus 85.45 cm, and 102.2 versus 93.45  cm, respectively. All *p* values were highly significant with 0.0001 (Table 2).

Age group showed the same profile, increasing proportion up to 59 years in urban as in rural areas. Age group 45–59 y made 37% (9.5 in rural and 27.5% in urban areas). But differences did not reach significant level (Table 3).

Female sex and the status married represented 60.2 and 81.4% of the sample, respectively, but with *p* value of 0.086 and 0.676 whereas almost half of the sample was unschooled (*p* < 0.0001) (Table 3).

**Figure 1:**
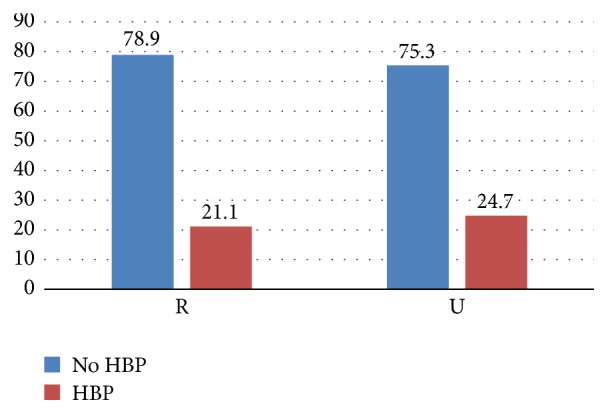
Blood pressure profile in rural and urban area.

HBP prevalence was 21.1 and 24.7%, respectively, in rural and urban setting (Figure 1).

Among blood pressure parameters, heart rate was lower in urban areas (78.8%) versus 81.25% in rural areas with a *p* value of 0.040. Systolic, diastolic, and mean arterial pressure were slightly higher in urban areas with, respectively, 151.23 mmHg, 94.62 mmHg, and 113.49 mmHg versus 150.94 mmHg, 94.10 mmHg, and 113.04 mmHg in rural areas. Corresponding *p* values were, respectively, 0.900, 0.675, and 0.753 (Table 4). Pulsed pressure was in the same level with 56.84 mmHg in rural areas and 56.61 mmHg in urban areas (*p* = 0.904).

Using logistic regression, age group was found to be a significant predictor for hypertension (*χ*^2^ = 139.13, df = 22, and *p* < 0.001) in rural areas. The OR were 2.60, 4.97, and 9.70 for 30–44 y, 45–59 y, and above 60 years old, respectively, with under 30 years as reference (Table 5).

Whereas in urban area age group, sex, and BMI were found as predictors for hypertension, hypertension increased in age group from 2.06 CI [95% 1.24–3.43] for 30–44 y to 7.25 CI [95% 4.00–13.13] for 60 y and above (*p* < 0.0001). Female sex was protective with OR of 0.45 IC [95% 0.29–0.71] and BMI showed an increase of hypertensive subjects with increasing BMI. OR for overweight was 1.54 IC [95% 1.04–2.27] and 2.67 IC [95% 1.64–4.36] for obesity.

## 4. Discussion

In the study with a large data sample, we first estimated HTN prevalence in rural and urban area and then conducted a logistic regression to look for predictors of HTN. To our knowledge it is the first time that community-based data are analyzed in such a way. Previous studies are either community-based in urban area [14] or from hospital based data.

### 4.1. Prevalence of Hypertension

HTN prevalence remains high in rural and urban setting with values above 20% as shown in Figure 1. There is more HTN in urban area than in rural area as found by many other authors [13, 19, 20]. Previous data [14] was conducted only in the urban city of Bamako. Compared to others parts of the world [5] our prevalence is relatively low. Trends over the time due to unavailability of data cannot be assessed as done for west African countries [21].

### 4.2. Hypertension in Rural Area

Our data suggest high prevalence of HTN as found in other rural areas in Africa [19, 22] but remains low compared to data from Ghana [23] with near twice our prevalence. This is opposed to data from rural areas in Sudan with near 16% [24].

Our high prevalence could be due to either a real increase in HTN prevalence due to changing in behaviors (less physical activity, diet modifications) or modification in the population.

Looking for predictive factors, we found that only age group and resting heart rate significantly predicted HTN, with OR for age group increases with increasing age from 1.05 to 3.4 as shown in Table 5. This also conforms to what is known about HTN and it increases with aging. Many factors are found to be associated with HTN such that in our study (Table 6) age and BMI were predictors for HTN as found by Abebe et al. [13] and Neupane et al. [25]. Fasting glucose [13] and income and educational level [23] were factors found by these authors.

### 4.3. Hypertension in Urban Area

The same finding of a high HTN prevalence was confirmed with our data. Our prevalence of 24.7% is practically equal to that found on data from the 2002 study which was conducted using the same WHO STEPwise approach and had involved only population of the urban city of Bamako.

In their systematic analysis Adeloye and Basquill [8] gave prevalence for countries of different regions Africa. Our prevalence was similar to that of sub-Saharan Africa as found by Adeloye and Basquill. Elsewhere, we see large differences in prevalence, but increase is constant [25, 26]. Some authors reported stable prevalence like in Switzerland [27], Turkey [28].

Opposed to finding in rural area, age, female sex, and body mass index were found to be predictive for HTN.

For age group the OR increases with age from 2.06 (30–44 years) to 7.25 (60 and more) which is similar finding in most studies [10, 13, 23] in Africa.

Female sex appeared to be protective in our study with an OR of 0.45.

Lastly BMI was the third predictive factor that we found with OR 1.54 for overweight and 2.67 of obese subjects. This increasing of HTN with increasing BMI has been described by many authors [13, 23].

### 4.4. Strength and Limits

Our data provide recent and community-based data on hypertension prevalence in rural and urban areas in Mali, using the WHO STEPwise survey strategy, and contribute to covering the need of such data.

We faced some difficulties which can be considered as limits. First, due to financial reason, lipid profile was not included in the 2013 STEPS-Survey. Second we also have been unable to take into account income and lastly comparison was difficult due to the lack of previous data.

## 5. Conclusion

Hypertension prevalence is high in rural and urban area and associated factors were age group in rural area and age group, female sex, and body mass index in urban area. Our data could be used as reference and serve to initiate more extensive studies permitting to get robust data for deciders. It is also important to find financial support to include lipid profile in the next STEPS-Survey.

## Figures and Tables

**Table 1:** 

Commune	Number of clusters	Type of area	Quartiers/villages
II (Bamako)	3	Urban	Bakaribougou, Médina coura; Niarela
III (Bamako)	3	Urban	Bamakocourabolibana, Dravelila, N'Tomicorobougou
VI (Bamako)	6	Urban	Banankabougou, Magnanbougou, Missabougou, Niamakoro, Sogoniko; Yirimadjo
Koulikoro	5	Urban	Katibougou, Kasso, Kolebougou, Koulikoroba, Koulikoro garre I
Kati central	3	Rural	Kati coura, Malibougou, Sébénicoro
Ouélessébougou	2	Rural	Fanicodiana, Tinkele
Ségou	4	Urban	Bagadadji, Darsalam, Mission, Sidosonicoura
Sikasso	4	Urban	Boula hameau, Lafiabougou koko, Quartuier résidentiel, Wayerma I

*Total*	*30*		

**Table 2:** Description of sociodemographic characteristics of 495 hypertensive subjects in the 2013 STEP survey in Mali.

Variables	Setting				Total		*p*
	Rural		Urban				
	*N*	%	*N*	%	*N*	%	
Age groups (years)							
16–29	14	2.8	41	8.3	55	11.1	0.845
30–44	28	5.7	97	19.6	125	25.3	
45–59	47	9.5	136	27.5	183	37.0	
60 and more	29	5.9	103	20.8	132	26.7	
Sex							
Male	39	7.9	158	31.9	197	39.8	0.086
Female	79	16.0	219	44.2	298	60.2	
Marital status							
Unmarried	8	1.6	32	6.5	40	8.1	0.676
Divorced	0	0.0	1	0.2	1	0.2	
Married	95	19.2	308	62.2	403	81.4	
Widowers	15	3.0	36	7.3	51	10.3	
Education level							
Level 0	79	16.0	165	33.3	244	49.3	<0.0001
Level 1	11	2.2	84	17.0	95	19.2	
Level 2	14	2.8	51	10.3	65	13.1	
Level 3	13	2.6	63	12.7	76	15.4	
Level 4	1	0.2	14	2.8	15	3.0	

**Table 3:** Description of continuous variables of 495 hypertensive subjects in rural and urban setting in Mali.

	Rural			Urban			Total			*p*
	Mean	*N*	SD	Mean	*N*	SD	Mean	*N*	SD	
Age	47.00	118	13.214	48.03	377	13.243	47.78	495	13.230	0.451
Weight (Kg)	68.50	116	13.305	75.47	376	16.510	73.83	492	16.076	<0.0001
Height (cm)	165.76	118	09.708	167.60	375	09.711	167.16	493	9.732	0.074
BMI	24.83	116	04.346	26.29	374	05.516	26.41	490	5.330	<0.0001
WC	85.48	114	11.819	92.79	368	14.123	91.06	482	13.952	<0.0001
HC	93.50	115	12.914	102.31	334	15.315	100.05	449	15.218	<0.0001
rWH	0.92	111	0.087	0.91	328	0.108	0.92	439	0.103	0.634
Glycemia	116.23	117	29.638	120.18	363	66.882	119.22	480	59.968	0.536

BMI: body mass index; WC: waist circumference; HC: hip circumference; rWH: waist-to-hip ratio.

**Table 4:** Blood pressure related parameters in the sample of 495 hypertensive subjects.

	Rural			Urban			Total			*p*
	Mean	*N*	SD	Mean	*N*	SD	Mean	*N*	SD	
SBP	150.94	118	22.734	151.23	377	21.630	151.16	495	21.875	0.900
DBP	94.10	118	13.193	94.62	377	11.310	94.49	495	11.774	0.675
HR	81.25	118	16.177	78.03	377	14.368	78.80	495	14.866	0.040
PP	56.84	118	17.421	56.61	377	18.224	56.67	495	18.019	0.904
mBP	113.04	118	14.862	113.49	377	12.939	113.38	495	13.408	0.753

SBP: systolic blood pressure; DBP: diastolic blood pressure; HR: heart rate; PP: pulsed pressure; mBP: mean blood pressure.

**Table 5:** Logistic regression predicting hypertension in rural setting.

Variables	*B*	S.E.	df	Sig.	OR	95% CI for OR	
						Lower	Upper
Age group: <30 years as reference			3	.000			
30–44	.955	.462	1	.039	2.60	1.05	6.42
45–59	1.603	.468	1	.001	4.97	1.99	12.42
60 and more	2.272	.535	1	.000	9.70	3.40	27.66
Marital status: single as reference			2	.904			
Married	.018	.544	1	.974	1.02	0.35	2.96
Widower	.220	.706	1	.755	1.25	0.31	4.97
Sex	−.811	.414	1	.050	0.44	0.20	1.00
Educational level: unschooled as reference			4	.595			
Level 1	−.336	.441	1	.446	0.71	0.30	1.70
Level 2	−.123	.403	1	.761	0.88	0.40	1.95
Level 3	.008	.426	1	.986	1.01	0.44	2.32
Level 4	−1.654	1.092	1	.130	0.19	0.02	1.63
Tobacco smoking	.042	.389	1	.914	1.04	0.49	2.24
Alcohol consumption	−.242	.713	1	.735	0.79	0.19	3.18
BMI: Underweight as reference			3	.887			
Normal weight	.395	.661	1	.550	1.49	0.41	5.43
Overweight	.537	.702	1	.445	1.71	0.43	6.78
Obesity	.411	.803	1	.608	1.51	0.31	7.28
WC: normal as reference			2	.211			
Overweight	.390	.350	1	.265	1.48	0.74	2.94
Obesity	.711	.408	1	.082	2.04	0.91	4.53
rWH: normal as reference			2	.054			
Overweight	.296	.431	1	.491	1.35	0.58	3.13
Obesity	1.066	.465	1	.022	2.90	1.17	7.23
Diabetes	.133	.383	1	.728	1.14	0.54	2.42
Resting HR > 90/min	1.551	.652	1	.017	4.72	1.32	16.94

**Table 6:** Logistic regression predicting hypertension in urban setting.

Variables	*B*	S.E.	df	Sig.	OR	95% CI for OR	
						Lower	Upper
Age group: <30 years as reference			3	.000			
30–44	.724	.259	1	.005	2.06	1.24	3.43
45–59	1.459	.261	1	.000	4.30	2.58	7.18
60 and more	1.981	.303	1	.000	7.25	4.00	13.13
Marital status: single as reference			3	.369			
Married	.224	.269	1	.404	1.25	0.74	2.12
Widower	.420	.408	1	.303	1.52	0.68	3.38
Divorced	−1.346	1.121	1	.230	0.26	0.03	2.34
Female sex	−.789	.228	1	.001	0.45	0.29	0.71
Educational level: unschooled as reference			4	.284			
Level 1	−.079	.209	1	.705	0.92	0.61	1.39
Level 2	−.030	.257	1	.907	0.97	0.59	1.61
Level 3	−.372	.223	1	.096	0.69	0.44	1.07
Level 4	−.622	.370	1	.093	0.54	0.26	1.11
Tobacco smoking	.087	.261	1	.738	1.09	0.65	1.82
Alcohol consumption	.083	.334	1	.803	1.09	0.57	2.09
BMI: Normal as reference			2	.000			
Overweight	.429	.200	1	.032	1.54	1.04	2.27
Obesity	.983	.249	1	.000	2.67	1.64	4.36
WC: normal as reference			2	.054			
Overweight	.451	.258	1	.080	1.57	0.95	2.60
Obesity	.665	.281	1	.018	1.95	1.12	3.37
rWH: normal as reference			2	.407			
Overweight	.199	.232	1	.391	1.22	0.77	1.92
Obesity	.334	.250	1	.182	1.40	0.86	2.28
Diabetes	.069	.225	1	.760	1.07	0.69	1.67
Resting HR > 90/min	.274	.476	1	.564	1.32	0.52	3.35
